# Alterations in Phospholipid Levels and Spatial Distribution in the Motor Cortex and Their Correlation with Motor Performance in an MPTP-Induced Parkinsonian Mouse Model

**DOI:** 10.3390/molecules31071175

**Published:** 2026-04-02

**Authors:** Morakot Sroyraya, Kunwadee Noonong, Prasert Sobhon, Tanapan Siangcham, Wanwisa Waiyaput, Veerawat Sansri, Kulathida Chaithirayanon, Pennapa Chonpathompikunlert

**Affiliations:** 1 Department of Anatomy, Faculty of Science, Mahidol University, Bangkok 10400, Thailand; morakot.sry@mahidol.ac.th (M.S.);; 2 School of Allied Health Sciences, Walailak University, Nakhon Si Thammarat 80160, Thailand; 3 Research Excellence Center for Innovation and Health Product (RECIHP), Walailak University, Nakhon Si Thammarat 80160, Thailand; 4 Faculty of Allied Health Sciences, Burapha University, Chonburi 20131, Thailand; 5 Office of Research Academic and Innovation, Faculty of Medicine Ramathibodi Hospital, Mahidol University, Bangkok 10400, Thailand; 6 Department of Basic Medical Science, Faculty of Medicine Vajira Hospital, Navamindradhiraj University, Bangkok 10300, Thailand; 7 Pre-Clinical and Clinical Research Service Unit (P2CRSU), Biodiversity Research Centre (BRC), Research and Development Group for Bio-Industries, Thailand Institute of Scientific and Technological Research (TISTR), Pathumthani 12120, Thailand

**Keywords:** Parkinson’s disease, MPTP, lipid alterations, polyunsaturated fatty acids, MALDI-MSI

## Abstract

Parkinson’s disease (PD) is a neurodegenerative disorder caused by the death of dopaminergic neurons in the substantia nigra pars compacta (SNc). Lipid metabolism, especially phospholipids, has been reported to be altered in PD. The purpose of this study is to investigate the temporal expression and spatial distribution of phospholipids in the motor cortex and striatum at different time points of PD using matrix-assisted laser desorption/ionization mass spectrometry imaging (MALDI-MSI) in a 1-methyl-4-phenyl-1,2,3,6-tetrahydropyridine (MPTP)-induced parkinsonian mouse model. Mice were injected with saline (NSS) or MPTP at two different time points to create acute and subacute models. Motor analysis was performed at 0, 3, 7, 14, and 21 days post-injection. Tyrosine hydroxylase (TH) staining revealed progressive damage of neurons in the substantia nigra compacta (SNc) and reduced striatal fibers in MPTP-treated animals. By using MALDI-MSI, we identified changes in phosphatidylcholine (PC) profiles in the brains of MPTP-treated animals. Polyunsaturated PCs, including PC 36:4 (16:0/20:4), PC 38:6 (16:0/22:6), and PC 40:8 (18:2/22:6), were decreased in the MPTP-treated groups. These reductions were time-dependent and were more pronounced in the subacute MPTP-treated group. The loss of dopamine neurons caused by MPTP may be associated with the selective loss of polyunsaturated PCs in brain membranes, indicating that lipid metabolism and membrane structural alterations may contribute to the pathology of PD.

## 1. Introduction

Parkinson’s disease (PD) is a common neurodegenerative disorder that mainly affects persons older than age 65 [1]. Parkinson’s disease results in progressive loss of motor control. The early onset symptoms typically include tremor, stiffness, slowing of motor processes (bradykinesia), and impaired postural stability [2]. The major motor symptoms of Parkinson’s disease (PD) arise from the death of dopaminergic neurons in the substantia nigra pars compacta (SNc), resulting in decreased dopaminergic input to the striatum and disruption of the striatal circuits that regulate motor function [3]. Similar to Alzheimer’s disease, PD has a distinctive set of neuropathological changes. In PD, there is heavy degeneration of dopaminergic neurons in the SNc. In addition, neurons in the SNc and other brain areas develop characteristic cytoplasmic inclusions called Lewy bodies. These mixed protein aggregates contain several proteins and protein fragments of interest, including alpha-synuclein, ubiquitin, parkin, synphilin-1, oxidized proteins, heat shock proteins, neurofilaments, and various proteasomal subunit components [2,4]. Dopaminergic neurons in the SNc cause a degenerative impact not only within the midbrain, but also in extra-axial regions, such as the putamen and caudate nucleus, responsible for control of movement; thus, muscle function is impaired [2,5].

Despite the presence of proteinaceous abnormalities that are known to be present in PD, lipid metabolism has also been shown to be increasingly involved in the disease. The brain is very rich in lipids, especially phospholipids, which account for about 6% of its dry weight [6]. Phospholipids and sphingolipids are essential components of neuronal membranes. They are important for the structure, signaling, and homeostasis of cells [7]. Increasing evidence indicates that phospholipid metabolism is disrupted in PD, with alterations observed across multiple lipid classes, including phosphatidylcholine (PC), phosphatidylethanolamine (PE), sphingomyelin (SM), and ceramides [8,9]. Mechanistically, phospholipid alterations in PD are linked to key disease-associated pathways, including mitochondrial dysfunction, lysosomal impairment, and abnormal lipid signaling. Several PD-related genes, such as LRRK2, PLA2G6, and glucocerebrosidase, are directly involved in lipid metabolism and membrane remodeling, further supporting the role of phospholipid dysregulation in disease pathogenesis [9]. In addition, interactions between α-synuclein and membrane phospholipids are critical for protein aggregation and toxicity, highlighting the interplay between lipid imbalance and α-synuclein-associated pathology in PD [8,10]. Recent studies have shown significant lipid remodeling in cortical and motor-related brain regions in PD, as revealed by mass spectrometry (MS)-based analyses. Alterations in phospholipids (e.g., PC, PE) and sphingolipids, particularly ceramides, have been consistently observed and are associated with motor dysfunction and cortical–motor circuit alterations [11]. These changes support the involvement of lipid-mediated inflammatory and apoptotic pathways in PD [11]. In addition to brain tissue, alterations in the cerebrospinal fluid (CSF) lipidome have also been reported in PD, further supporting the presence of systemic lipid dysregulation [12].

Mass spectrometry (MS) is recognized as one of the most powerful and sensitive analytical techniques for lipid analysis [13,14]. Liquid chromatography–mass spectrometry (LC-MS) and gas chromatography–mass spectrometry (GC-MS) are sensitive and effective methods for identifying and quantifying many classes of lipids [15,16,17]. Nonetheless, these methods require extraction from tissues, thereby destroying the in situ intact distributions of lipid species. Matrix-assisted laser desorption/ionization mass spectrometry imaging (MALDI-MSI) is an alternative method and a powerful analytical technique for detecting the localization of various biomolecules in tissue sections without the need for immunohistochemical stainings [18,19,20]. It enables spatially resolved analysis of lipid alterations in brain tissues and can detect early molecular changes preceding histopathological damage [21,22,23]. For example, in a transient global ischemia model, MALDI-MSI detected early alterations in PC species prior to neuronal loss [21]. Beyond neurodegeneration, MALDI-MSI has been applied in glioma and human brain studies, where lipid signatures, including phosphatidic acid (PA) and diacylglycerol (DAG), are associated with tumor behavior and white matter abnormalities [22,23]. Notably, MALDI-MSI has revealed region-specific lipid alterations in periventricular white matter lesions, supporting its utility in studying disease-associated lipid remodeling in the brain [23]. Although lipid alterations in PD have been reported in cortical regions and the CSF using MS-based analyses [11,12], the spatial distribution of phospholipids in the cortex remains poorly characterized. Therefore, this study aims to investigate alterations in phospholipid composition and their spatial distribution in the motor cortex of an MPTP-induced parkinsonian mouse model using MALDI-MSI.

## 2. Results

### 2.1. Effects of MPTP on Body Weight and Motor Coordination

Following the injection of MPTP, the weight was measured, and motor functions, including narrow beam and grid walk tests, of all mice were performed at days 0, 3, 7, 14, and 21. To assess that MPTP did not have an effect on eating behavior, the animal weights were measured. The results showed that the average weights of acute and subacute MPTP-injected animals were not significantly different when compared with those in the NSS-injected group (Table 1), indicating that MPTP did not impair feeding behavior.

**Figure 1:**
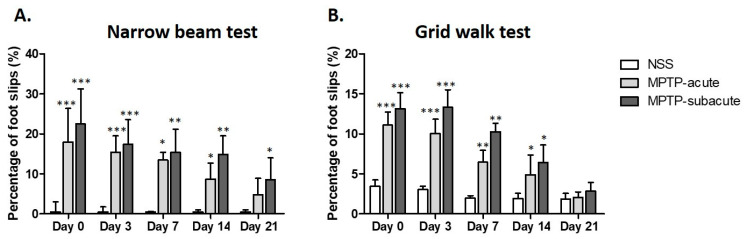
Motor performance assessed by (**A**) narrow beam test and (**B**) grid walk test in NSS, MPTP-acute, and MPTP-subacute groups over time (Day 0–21). Data are presented as median with interquartile range (IQR). Statistical significance was assessed using the Kruskal–Wallis test followed by Dunn’s multiple comparisons test with Bonferroni correction. Adjusted *p*-values were used for multiple comparisons. Significant differences compared with the NSS group are indicated as *, **, and *** based on adjusted *p*-values.

The narrow beam and grid walk tests (Figure 1A,B) were used to check motor coordination and balance. In the narrow beam test, latency time was temporarily elevated in the acute MPTP-injected group on day 0, but no significant differences were observed among groups from day 3 to day 21, indicating recovery of gross motor function. In contrast, balance deficits evaluated using the percentage of foot slips exhibited greater sensitivity and prolonged duration. On days 0, 3, 7, and 14, both acute and subacute MPTP-injected mice demonstrated markedly increased percentages of foot slips compared with control subjects. The subacute MPTP-injected group consistently exhibited greater impairment than the acute group, indicating more severe motor dysfunction, and the percentage of foot slips in the subacute group remained significantly elevated on day 21 (Figure 1A). Similarly, in the grid walk test (Figure 1B), MPTP-treated mice exhibited markedly elevated percentages of foot slips during the early time points (days 0–7), especially within the subacute MPTP-treated group. These impairments gradually ameliorated over time, exhibiting no significant differences at subsequent stages (day 21), indicative of partial functional recovery.

### 2.2. TH Immunoreactivity in the Substantia Nigra and Striatum

**Figure 2:**
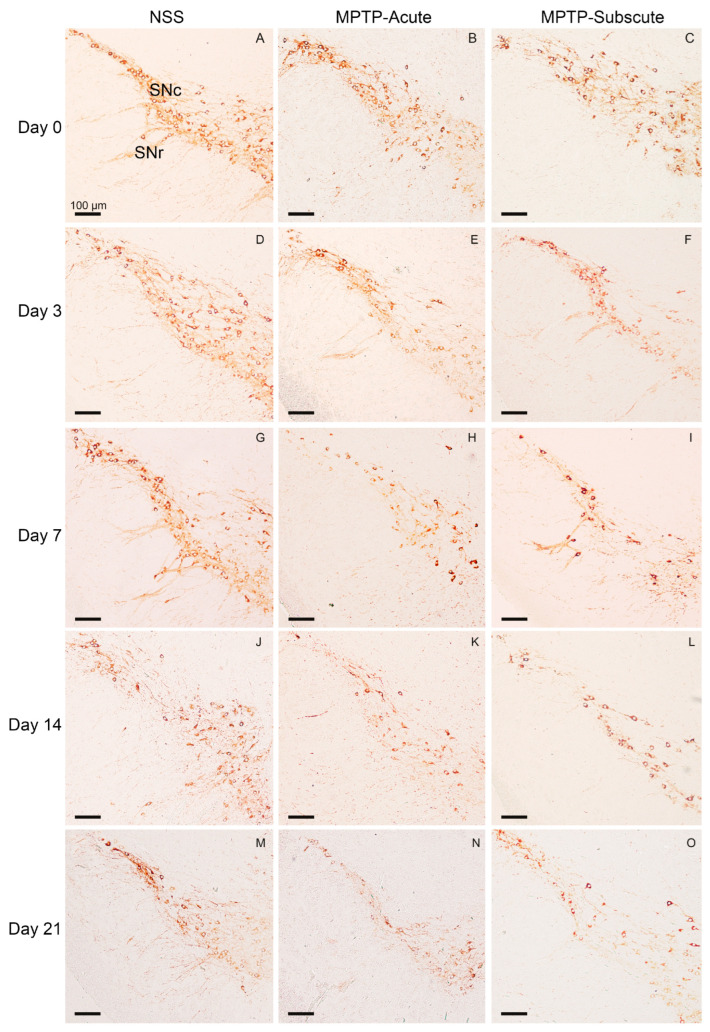
Immunoperoxidase staining of TH in the substantia nigra of mice. (**A**,**D**,**G**,**J**,**M**) Representative microphotographs showing TH-positive neurons in the substantia nigra pars compacta (SNc) and fiber in the substantia nigra pars reticulata (SNr) of the control group injected IP with NSS at days 0, 3, 7, 14, and 21, respectively. (**B**,**E**,**H**,**K**,**N**) Microphotographs showing the mouse brain of acute injected IP with MPTP (MPTP-Acute) at days 0, 3, 7, 14, and 21, respectively. (**C**,**F**,**I**,**L**,**O**) Microphotographs showing the mouse brain of subacute injected IP with MPTP (MPTP-Subacute) at days 0, 3, 7, 14, and 21, respectively. Scale bar of all micrographs: 100 μm.

**Figure 3:**
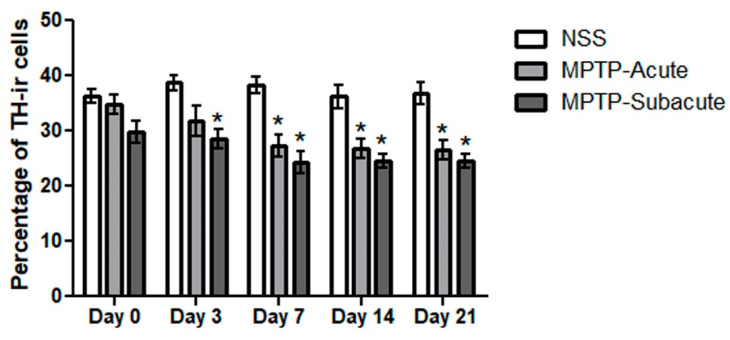
TH-positive dopaminergic neurons in substantia nigra pars compacta (SNc), confirming that exposure to MPTP led to significant neurodegeneration in acute and subacute MPTP-induced mice. Data are expressed as the means ± SEM (*n* = 5 each). * *p* < 0.01 control-NSS versus MPTP-Acute or MPTP-Subacute groups. NSS, normal saline; MPTP-Acute, mice injected with acute MPTP; MPTP-Subacute, mice injected with subacute MPTP.

After behavioral tests, the animals were sacrificed at days 0, 3, 7, 14, and 21. The number of dopaminergic neurons was evaluated by TH staining in the substantia nigra (Figure 2 and Figure 3). TH was highly expressed in the cytoplasm of dopaminergic neurons in the substantia nigra pars compacta (SNc), and fiber projections were seen in the substantia nigra pars reticulata (SNr) (Figure 2). The number of TH-positive cells in control mice injected IP with NSS was not different from day 0 to day 21 after NSS injection (Figure 2 and Figure 3). However, the number of TH-positive neurons in the SNc of acute MPTP injection was reduced from day 3 (Figure 2 and Figure 3). The number of TH-positive cells in the subacute MPTP injection was significantly reduced from day 3. Moreover, TH-positive cells were dramatically reduced in subacute MPTP injected from day 7 to day 21 (Figure 2 and Figure 3).

**Figure 4:**
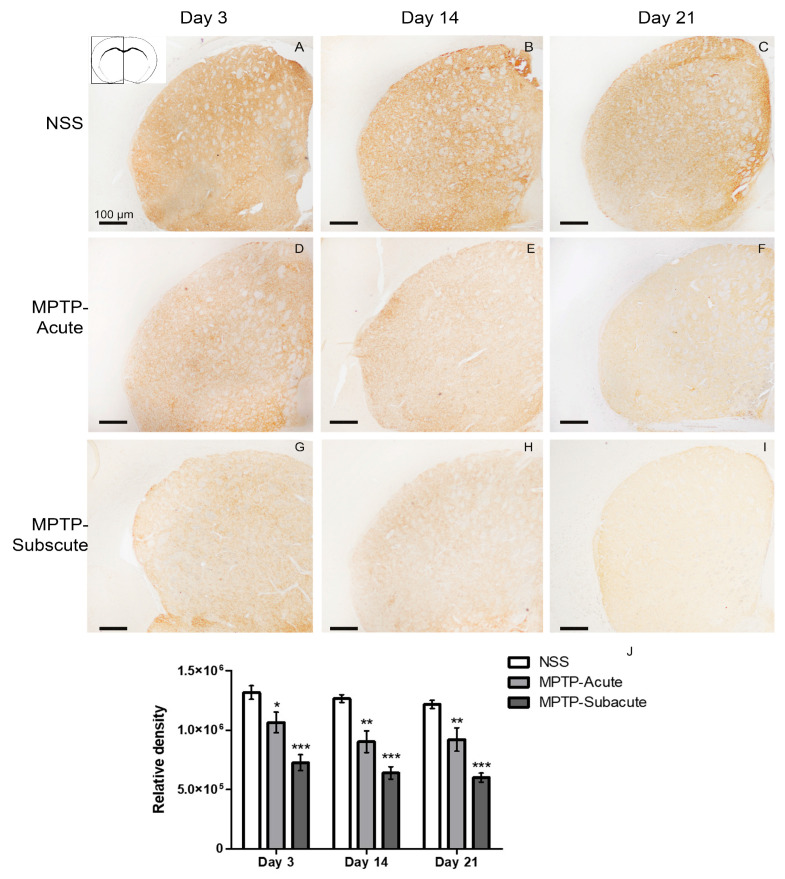
Immunoperoxidase staining of TH in the striatum and cortex of the mice. (**A**–**C**) Representative microphotographs showing TH-positive fibers in the striatum of the control group injected IP with NSS at days 3, 14, and 21, respectively. The inset of (**A**) shows the anatomical location of the striatum, and the outlined area is where TH immunostaining was focused. (**D**–**F**) Microphotographs showing the mouse striatum of acute injected IP with MPTP (MPTP-Acute) at days 3, 14, and 21, respectively. (**G**–**I**) Microphotographs showing the mouse striatum of subacute injected IP with MPTP (MPTP-Subacute) at days 3, 14, and 21, respectively. Scale bar of all micrographs: 100 μm. (**J**) Quantitative analysis of TH-positive staining, expressed as relative density, demonstrated a slight reduction in the acute group and a significant reduction in the subacute group at day 3, followed by a marked decrease in both groups at days 14 and 21. Data are presented as the mean ± SEM. * *p* < 0.05, ** *p* < 0.01, *** *p* < 0.001 compared to the NSS group.

Consistent with these findings, TH immunoreactivity in the striatum was evaluated. At day 0, no significant differences were observed among the three groups. At day 3 after NSS or MPTP administration, the results indicate that in the acute MPTP-treated group, fiber density is slightly reduced, while the subacute MPTP-treated group shows a more pronounced reduction. Day 7, day 14, and day 21 show the same result that both acute and subacute MPTP-treated groups have significantly decreased (Figure 4).

### 2.3. Phospholipid Distribution and Temporal Profiles Revealed by MALDI-MSI

**Figure 5:**
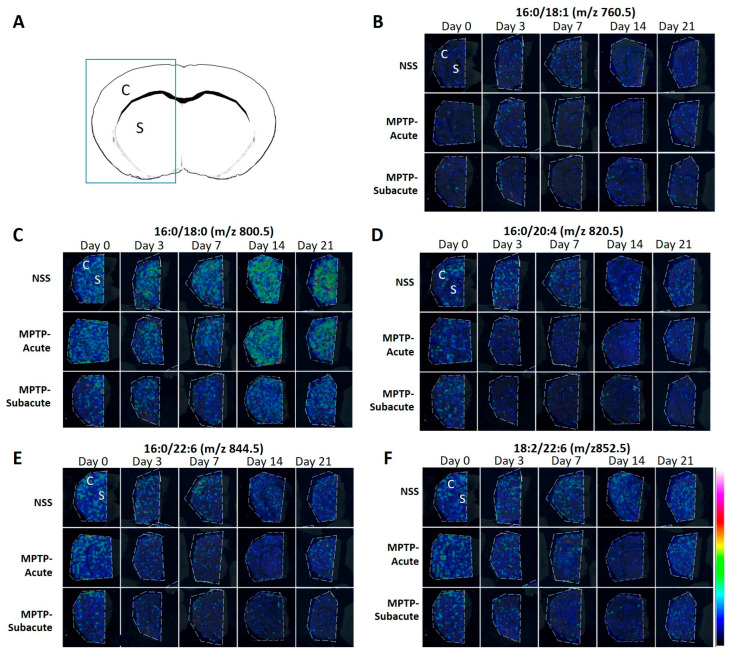
The distribution of each lipid species in the motor cortex (C) and striatum (S) was revealed by MALDI-MSI. (**A**) Schematic representation of a coronal brain section showing the motor cortex (C) and striatum (S). The boxed area represents the region analyzed by MALDI-MSI. (**B**–**F**) Representative ion images of the PC species (**B**) PC 34:1 (16:0/18:1, *m*/*z* 760.5), (**C**) PC 34:0 (16:0/18:0, *m*/*z* 800.5), (**D**) PC 36:4 (16:0/20:4, *m*/*z* 820.5), (**E**) PC 38:6 (16:0/22:6, *m*/*z* 844.5), and (**F**) PC 40:8 (18:2/22:6, *m*/*z* 852.5) in the NSS (control), mice injected with acute MPTP (MPTP-acute), and mice injected with subacute MPTP (MPTP-subacute) groups on the indicated days (*n* = 5). The color scale represents ion intensity.

To investigate the molecular changes that may be associated with dopaminergic neuron death, lipid distribution and lipid changes were analyzed using MALDI-MSI in the motor cortex and striatum at days 0, 3, 7, 14, and 21. A representative mass spectrum with annotated peaks is shown in Supplementary Figure S1. Five high-abundance mass peaks were selected for analysis and tentatively assigned to PC species based on their *m*/*z* values and comparison with previously reported data (Table S1) [24,25,26]. These annotated peaks were consistently observed across samples and were used for subsequent interpretation of lipid alterations, whereas lower-intensity peaks were not further analyzed. The selected ions were tentatively assigned to phosphatidylcholine (PC) species as follows: *m*/*z* 760.5 (PC 34:1, 16:0/18:1), *m*/*z* 800.5 (PC 34:0, 16:0/18:0), *m*/*z* 820.5 (PC 36:4, 16:0/20:4), *m*/*z* 844.5 (PC 38:6, 16:0/22:6), and *m*/*z* 852.5 (PC 40:8, 18:2/22:6). The spatial distributions of these lipid species, as determined by MALDI-MSI, are shown in Figure 5.

**Figure 6:**
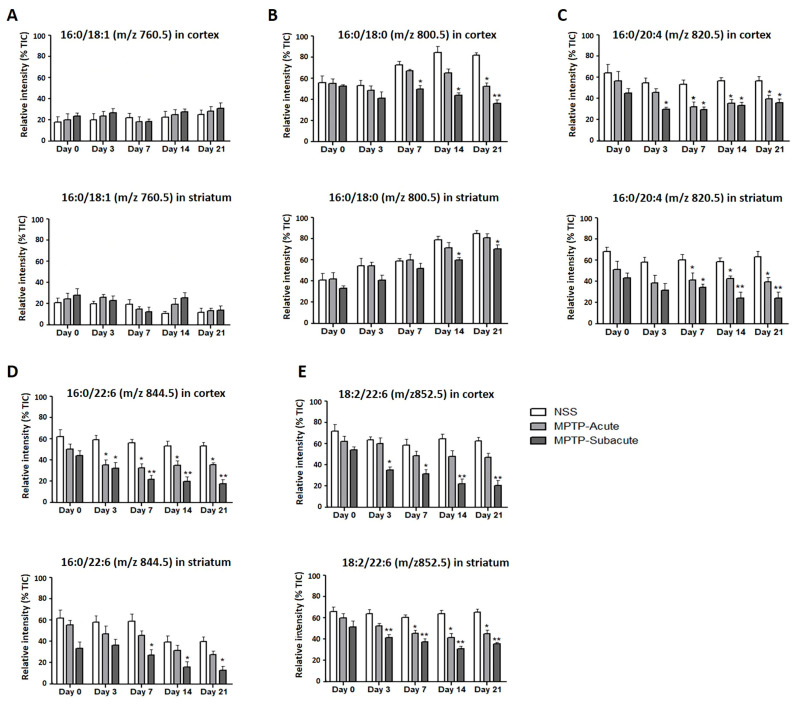
(**A**–**E**) Relative intensities (% TIC) of representative PC species, including *m*/*z* 760.5 (16:0/18:1), *m*/*z* 800.5 (16:0/18:0), *m*/*z* 820.5 (16:0/20:4), *m*/*z* 844.5 (16:0/22:6), and *m*/*z* 852.5 (18:2/22:6), measured in the cortex and striatum. Signal intensities were normalized to the total ion current (TIC) and expressed as relative intensity (% TIC) for comparison of lipid abundance between groups. Data are expressed as the mean ± SEM. * *p* < 0.05, ** *p* < 0.01 compared with the NSS group.

The relative intensities (% TIC) of specific PC species were determined in the motor cortex and striatum at various time points. In the cortex, the PC 34:1 (16:0/18:1, *m*/*z* 760.5) species was unchanged within all groups and time points in both the cortex and striatum (Figure 6A). The PC 34:0 (16:0/18:0, *m*/*z* 800.5) species was significantly decreased in the cortex of subacute MPTP-treated animals over time, especially from days 14 to 21 (Figure 6B). Some of the polyunsaturated species, such as PC 36:4 (16:0/20:4, *m*/*z* 820.5), PC 38:6 (16:0/22:6, *m*/*z* 844.5), and PC 40:8 (18:2/22:6, *m*/*z* 852.5), decreased in both acute and subacute MPTP-treated animals, and the decrease was significant from day 7 (Figure 6C–E). The decrease was especially noticeable in the subacute MPTP-treated group compared to the acute MPTP-treated group.

## 3. Discussion

The present study demonstrates that MPTP-induced parkinsonism is associated with degeneration of dopaminergic neurons in conjunction with altered phospholipid metabolism and structural organization within the brain. Similar to previous reports [27,28], in this study, administration of MPTP caused a significant decrease in the number of TH-positive neurons in the SNc and a decrease in fiber density in the striatum. The result confirms that the nigrostriatal pathway has been disrupted, which is a sign of PD [29]. In addition, following MPTP treatment, there was an increased number of foot slip errors. Mice injected with the subacute MPTP group had more severe and sustained deficits than those injected with the acute MPTP group, suggesting a greater degree of neurodegeneration and a more severe disease-like state following subacute treatment.

We investigated the time-course profiles of motor function parameters that were differentially affected in mice injected with MPTP and elucidated the types of motor dysfunction responsible for their changes. Although the latency time to pass through on a narrow beam, which is an index of general locomotor capability, was recovered on day 3 after injection, the number of foot slip errors that reflect motor coordination function persisted for a longer duration after injection. This result may indicate that gross motor skills, such as movement speed, may improve or be compensated for over time, while fine motor coordination and balance remain inadequate at the late time point [30]. Changes in foot slip errors are consistent with previous reports of movement abnormalities in other PD animal models [31,32].

The present study further demonstrates a spatiotemporal relationship between dopaminergic neuron degeneration and lipid alterations in the MPTP-intoxicated PD model. By using TH staining, the results showed that the number of neurons in the SNc and the relative density of TH-containing fibers in the striatum were decreased in the subacute MPTP model. Dopaminergic innervation to the striatum was reduced, possibly due to the loss of dopaminergic neurons in the SNc that project to the striatum, leading to impaired synaptic transmission and motor dysfunction. The association between anatomical lesions and behavioral symptoms, such as the elevation of foot slip errors, also supports the sensitivity of coordination-based measurements.

Consistent with these anatomical and physiological changes, MALDI-MSI results revealed alterations in membrane molecular composition and structure, particularly in PC species. A direct quantitative correlation between MSI-derived molecular features and biochemical measurements was not performed due to differences in sample preparation and data acquisition. Nevertheless, the overall trends observed in MSI were consistent with the biochemical data. This limitation should be considered when interpreting the results. It should be noted that other abundant phospholipid classes, including phosphatidylserine (PS), PE, and SM, were not prominently detected in the present MALDI-MSI analysis. This may be related to the analytical conditions used, as MALDI-MSI in positive ion mode tends to preferentially detect certain lipid classes [24]. In addition, the high abundance of PC may cause ion suppression, which can reduce the detection sensitivity of other phospholipids. PE and PS also generally show lower ionization efficiency in positive ion mode and are more effectively detected in negative ion mode.

Notably, the amounts of saturated PC 34:0 and polyunsaturated PCs, including arachidonic acid (ARA)-containing PC 36:4 and docosahexaenoic acid (DHA)-containing PC 38:6 and PC 40:8, decreased in a time-dependent manner. These changes were more pronounced in MPTP-injected mice, particularly in the subacute model and in the striatum, a key brain region affected in PD. The decrease in polyunsaturated phosphatidylcholines (PUFA-PCs) may be associated with oxidative stress. Although PUFAs such as ARA and DHA are required for membrane fluidity, synaptic activity, and various cellular signaling cascades [33,34], the multiple unsaturated bonds make them highly susceptible to ROS. ROS has the potential to initiate lipid peroxidation, specifically damaging phospholipids that contain unsaturated fatty acids in neuronal membranes, resulting in membrane dysfunction [35]. Lipid peroxidation of neuronal membranes in the case of sublethal damage can serve as a marker of damage to specific neurons [36,37]. However, these oxidative stress-related mechanisms were not directly assessed in the present study. Therefore, further investigations are required to evaluate oxidative stress markers to confirm these proposed mechanisms.

Previous studies indicated that the balance between DHA and ARA is also important to modulate neuroinflammation and neuroprotection. The consumption of α-linolenic acid can cause an increase in DHA levels in the brain, decrease neuroinflammation, and improve behavioral outcomes after traumatic brain injury [38]. Higher DHA has been shown to decrease inflammatory signaling cascades [34], and DHA-derived oxylipins have potential anti-inflammatory effects [39]. In addition, higher DHA has been shown to improve behavioral outcomes and to decrease damage in spinal cord and ischemic injury models [40,41]. Moreover, an optimal balance between ARA- and DHA-derived lipid species is required to regulate inflammatory and oxidative pathways, which are exacerbated by overactivation of ARA lipid signaling [42]. The selective decrease in DHA- and ARA-containing PCs shown in this study may be a result of oxidative degradation and/or an imbalance of lipid signaling.

Furthermore, previous studies have suggested that a decrease in PUFA-containing PCs may result from membrane turnover during neuroinflammation [43,44]. During inflammation, PUFAs may shift from membrane phospholipids to storage lipids such as triacylglycerol (TAG) [43]. This then decreases the amount of PUFA in neuronal membranes. In parallel with this mechanism, the MALDI-MSI analysis in the current study revealed region-specific lipid alterations, showing a more pronounced reduction in PUFA-containing PCs in the striatum, which is consistent with the pattern of dopaminergic degeneration observed by TH staining. Importantly, lipid changes were more pronounced in the subacute MPTP-intoxicated model, suggesting that a longer toxin exposure results in greater lipid alterations. On the other hand, the relative stability of PC 34:1 suggests that PUFA-containing phospholipids are selectively vulnerable, rather than all membrane lipids. Moreover, PUFAs in the brain of MPTP-injected mice may interact with α-synuclein [6,45,46]; however, this interaction was not directly examined in the present study. It has been reported that interaction of α-synuclein and free PUFAs initially forms soluble forms and then aggregates into larger insoluble complexes with higher molecular weight, while monounsaturated fatty acids had no effects [47,48]. Formation of these PUFA-mediated protein aggregations increases cytotoxicity and may modulate neurodegeneration [49]. However, several limitations should be considered when interpreting these results. Lipid identification was performed based on *m*/*z* matching with previously reported data and was not confirmed by tandem mass spectrometry (MS/MS) or comparison with lipid databases. Therefore, the reported lipid species should be considered as putative identifications. In addition, multiple adduct forms ([M + H]^+^, [M + Na]^+^, and [M + K]^+^) may be detected for the same lipid species in MALDI-MSI, which may complicate peak interpretation. Here, representative ion signals were selected based on signal intensity, and a systematic comparison across all possible ion forms was not performed. Future studies incorporating MS/MS validation and adduct-specific spatial analysis would further strengthen lipid identification and interpretation.

Despite these limitations, this study demonstrates the potential of integrating TH immunohistochemistry with MALDI-MSI to investigate pathological changes in the brains of PD animal models. Further studies are needed to elucidate the mechanisms linking lipid metabolism with PD-related oxidative stress, neuroinflammation, and α-synuclein interactions, as well as to evaluate the therapeutic potential of lipid regulation.

## 4. Materials and Methods

### 4.1. Chemicals

A DAB kit, anti-tyrosine hydroxylase (TH), and horseradish peroxidase (HRP)-conjugated secondary antibody were purchased from Thermo Fisher Scientific Inc., Waltham, MA, USA. MPTP hydrochloride (HY-15608) was purchased from MedChemExpress (Monmouth Junction, NJ, USA). Bouin used as a fixative was obtained from Bio-optica (Milano, Italy). Matrix 2,5-dihydroxybenzoic acid (DHB) was purchased from Bruker Daltonics (Bremen, Germany). The matrix solvents trifluoroacetic acid (TFA) and methanol were purchased from Merck (Darmstadt, Germany) and RCI Labscan Limited (Bangkok, Thailand), respectively. All solvents used for mass spectrometry were HPLC grade.

### 4.2. Animals and Tissue Preparation

Twenty-five young adult male C57BL/6 mice aged 8–10 weeks (22–27 g) were used per group. Minitab Statistical Software’s Power and Sample Size tools (Minitab, LLC, State College, PA, USA; Version 16) were used to calculate the sample size. C57BL/6 mice were chosen in this experiment because this strain is susceptible to MPTP induction and exhibits PD-like symptoms [50,51]. The mice were obtained from the National Laboratory Animal Center, Mahidol University, Salaya Campus, Thailand. The experimental procedures followed the animal care criteria outlined by the Faculty of Science, Mahidol University. The research protocol was approved by the Faculty of Science, Mahidol University Animal Care and Use Committee, Thailand (No. MUSC58-017-332). The animals were randomly housed 5 per cage for 7 days to acclimatize in a temperature-controlled room with a 12/12-h light/dark cycle. Food and water was provided ad libitum. The animals were randomly divided into 3 groups, each with 25 animals: Group I (control mice) received 4 intraperitoneal (IP) injections of normal saline (NSS) (0.1 mL/0.1 kg) at 2-h intervals; Group II (acute MPTP-induced mice) received 4 IP injections of MPTP solution (15 mg/kg body weight per injection) in a volume of 1 mL/100 g body weight, at 2-h intervals; and Group III (subacute MPTP-induced mice) received 5 IP injections of MPTP solution (25 mg/kg body weight per injection) at 24-h intervals [52,53,54]. Motor function assessments were performed after 2 h (day 0), day 3, day 7, day 14, and day 21 after the last injection. Five animals per group were euthanized after motor function tests within 30 min using IP injection of 50 mg/kg pentobarbital and the brains were removed immediately. After that, the right and left hemispheres were cut to separate along the sagittal plane. The left hemisphere was immediately preserved in Bouin’s solution (pH 7.4) for 72 h, subsequently processed, and embedded in a paraffin block for immunohistochemical staining. The right hemisphere was immediately frozen in powdered dry ice and then stored at −80 °C until use for lipid analysis by using MALDI-MSI.

### 4.3. Motor Function Assessments

Narrow beam test: The narrow beam test [55] was used to check vestibulomotor function and coordination. The animals were trained to cross the narrow beams (L75 cm × W1 cm × H100 cm) to reach an enclosed platform. Videos and the time that mice took to cross the beam from one end to the platform (latency time) were recorded. Foot slips were recorded as an indicator of motor coordination and balance and expressed as a percentage of foot slips relative to the total number of steps.

Grid walk test: The grid walking task was employed to test for deficits with motor coordination, especially problems with limb placement while walking. In this test, mice were put on an elevated aluminum grid platform (60 × 40 cm, grid spacing 2.5 × 2.5 cm) that was 100 cm above the floor. Hindlimb slips through the grid openings were recorded as an indicator of motor dysfunction and expressed as a percentage of total hindlimb steps. To keep animals safe, a protective net was put up about 30 cm below the platform to prevent injuries from falling off.

### 4.4. Immunohistochemical Staining

Immunohistochemical staining for TH was utilized to visualize and quantify dopaminergic neurons in the substantia nigra pars compacta (SNc) and motor cortex. After 24 h of fixation, the brain tissues were washed with PBS 3 times for 5 min each. The brains went through a series of graded ethanol (70%, 80%, 90%, 95%, and 100% EtOH) before being moved to xylene. After that, the tissues were placed in paraffin. The Leica RM2235 rotary microtome (Leica Microsystems, Nussloch, Germany) cut all of the tissue sections to a thickness of 5 μm. Embedded tissues were trimmed and sectioned at bregma 0 ± 0.5 mm to acquire regions of the motor cortex. The embedded tissue from the posterior region was trimmed and sectioned at bregma, approximately −2.12 mm, to isolate areas of the SNc. After sectioning, the embedded sections were deparaffinized with xylene and rehydrated in 100%, 95%, 90%, 80%, and 70% ethanol, respectively. The sections were incubated with 70% ethanol saturated with lithium carbonate to abolish picric acid from the tissues. The endogenous peroxidase in the tissue were eliminated by incubating in 3% H_2_O_2_ in 100% methanol for 45 min. After being washed with 0.1 M PBST 3 times, the sections were incubated in 1% glycine in 0.1 M PBST for 15 min and then incubated with blocking solution containing 4% normal goat serum in 0.1 M PBST pH 7.4 for 2 h. The sections were incubated with polyclonal antibody TH at a dilution of 1:1000 at 4 °C overnight. The sections were washed with 0.1 M PBST 3 times followed by incubation with secondary antibodies, HRP-conjugated goat anti-rabbit IgG, at a dilution 1:5000 for 2 h. After that, DAB containing H_2_O_2_ was applied onto the tissues to develop the color for 10 min. The reaction was stopped by rinsing with tap water. The sections was counterstained with Mayer’s hematoxylin, mounted with Permount mounting medium, and then observed and photographed under a Nikon E600 microscope (Nikon Corporation, Tokyo, Japan) with Nikon digital DXM1200 camera (Nikon Corporation, Tokyo, Japan). The number of TH-positive cells was quantified by counting all labeled neurons in the SNc. In the striatum, TH-positive fiber density was evaluated using ImageJ software (National Institutes of Health, Bethesda, MD, USA; Version 1.42q), and the mean pixel intensity of TH-positive staining was measured. For each animal, measurements from five sections were averaged to obtain a single representative value, with each animal considered the experimental unit to avoid pseudo-replication.

### 4.5. Tissue Preparation and MALDI-MSI

Frozen brains were cut to 20 µm thickness by using a Leica CM1850 cryostat microtome (Leica Microsystems, Nussloch, Germany) at −20 °C. The sections were fixed onto an indiumtin-oxide (ITO)-coated glass slide (Bruker Daltonics). The matrix was DHB, which was uniformly spread over the tissue sections at a concentration of 50 mg/mL in a methanol:ultrapure water solution (7:3, *v*/*v*) with 0.1% trifluoroacetic acid (TFA). A MALDI-TOF mass spectrometer (ultrafleXtreme, Bruker Daltonics, Bremen, Germany) was used to analyze the brain sections. Mass spectra were acquired in positive ion linear mode, and external calibration was performed using Peptide Calibration Standard II (Bruker Daltonics, Bremen, Germany), covering a mass range of *m*/*z* 700–3500. For imaging analysis, spectra were collected in a mass range of *m*/*z* 500–1300, with a laser operating at 200 Hz, and 50 laser shots were accumulated per measurement point. The spatial resolution was set to 150 μm per pixel. Laser energy was optimized to maximize signal sensitivity during imaging analysis. FlexControl software (Bruker Daltonics, Bremen, Germany; Version 3.0) was used to perform raster scanning of the tissue sections, and 2D image reconstruction was carried out using FlexImaging 3.0 software (Bruker Daltonics). The mass spectra were normalized to the total ion current (TIC), and signal intensities were expressed as relative intensity (% TIC) based on integrated peak areas of selected *m*/*z* values for quantitative comparison of lipid abundance between groups. After MALDI-MSI analysis, the same tissue sections were cleaned with 100% methanol and then stained with hematoxylin and eosin (H&E).

### 4.6. Statistical Analysis

Behavioral assessments were conducted longitudinally in the same animals prior to sequential sacrifice for tissue collection; therefore, the effective sample size decreased over time. The sample size for behavioral analysis was *n* = 25, 20, 15, 10, and 5 per group at days 0, 3, 7, 14, and 21, respectively. This design enabled longitudinal behavioral assessment while minimizing the total number of animals used. In contrast, histological and MALDI-MSI analyses were performed using *n* = 5 mice per group at each time point, with each animal considered the experimental unit. Normality of data distribution was assessed using the Shapiro-Wilk test, and homogeneity of variances was evaluated using the Brown–Forsythe test. Behavioral data were not normally distributed; therefore, non-parametric analysis was performed using the Kruskal-Wallis test followed by Dunn’s multiple comparisons test with Bonferroni correction. Histological and MALDI-MSI data were found to follow a normal distribution and are presented as the mean ± SEM. For comparisons among groups at each time point, statistical tests were selected based on data distribution. A *p*-value < 0.05 was considered statistically significant, and adjusted *p*-values were used for multiple comparisons. Statistical analyses were performed using SPSS version 16.0 (SPSS, Cary, NC, USA), and graphs were generated using GraphPad Prism (version 5.0, GraphPad Software, San Diego, CA, USA).

## 5. Conclusions

In the mice model, it was revealed that progressive loss of dopaminergic neurons and fine motor coordination and balance was more pronounced in the subacute model. MALDI-MSI analysis revealed dynamic changes in the levels of saturated and polyunsaturated PC species in the MPTP-induced parkinsonian mouse model. Importantly, the expression levels of specific phospholipid species containing PUFAs, such as PC 36:4, PC 38:6, and PC 40:8, were decreased in the PD brain at different times after MPTP injection, while other lipid species, such as PC 34:1, were constant. The expression levels of certain lipids are consistent with the motor function deficits in PD. Our data supports the importance of lipid biology in PD, and using MALDI-MSI, we demonstrate that lipids are changing in the PD brain and that these changes are associated with important physiological outcomes.

## Figures and Tables

**Table 1:** Body weight of mice following NSS or MPTP administration at different time points.

Days After NSS or MPTP Injection	Average Weight (g)		
	NSS-Treated Group	Acute MPTP-Treated Group	Subacute MPTP- Treated Group
0	22.75 ± 0.88	23.57 ± 1.04	23.16 ± 0.63
3	23.14 ± 1.10	25.17 ± 1.18	24.22 ± 0.89
7	24.13 ± 1.39	25.73 ± 1.21	24.45 ± 0.92
14	25.70 ± 1.64	27.14 ± 1.08	26.82 ± 1.77
21	26.53 ± 1.23	26.45 ± 0.65	26.37 ± 1.16

## Data Availability

The data presented in this study are available on request from the corresponding author due to the large size and complexity of the raw mass spectrometry imaging datasets.

## Supplementary Materials

The following supporting information can be downloaded at: https://www.mdpi.com/article/10.3390/molecules31071175/s1, Figure S1: Representative MALDI-MSI spectra of phosphatidylcholine (PC) species at Day 7; Table S1: Putative identification of phosphatidylcholine (PC) species based on observed *m*/*z* values in MALDI-MSI analysis.

## Abbreviations

The following abbreviations are used in this manuscript:

DHB	2,5-Dihydroxybenzoic acid
TFA	Trifluoroacetic acid
IP	Intraperitoneal
C	Cortex
S	Striatum
SNc	Substantia nigra pars compacta
SNr	Substantia nigra pars reticulata
PUFAs	Polyunsaturated fatty acids
PCs	Phosphatidylcholines
