# Supplementary material for: Alterations in Phospholipid Levels and Spatial Distribution in the Motor Cortex and Their Correlation with Motor Performance in an MPTP-Induced Parkinsonian Mouse Model
Source: Molecules. 2026 Apr 2;31(7):1175. doi: 10.3390/molecules31071175 (PMC13075138; doi:10.3390/molecules31071175)
Supplement: Supplementary file 1 [file molecules-31-01175-s001.zip › molecules-4203490-supplementary.pdf]

Supplementary data

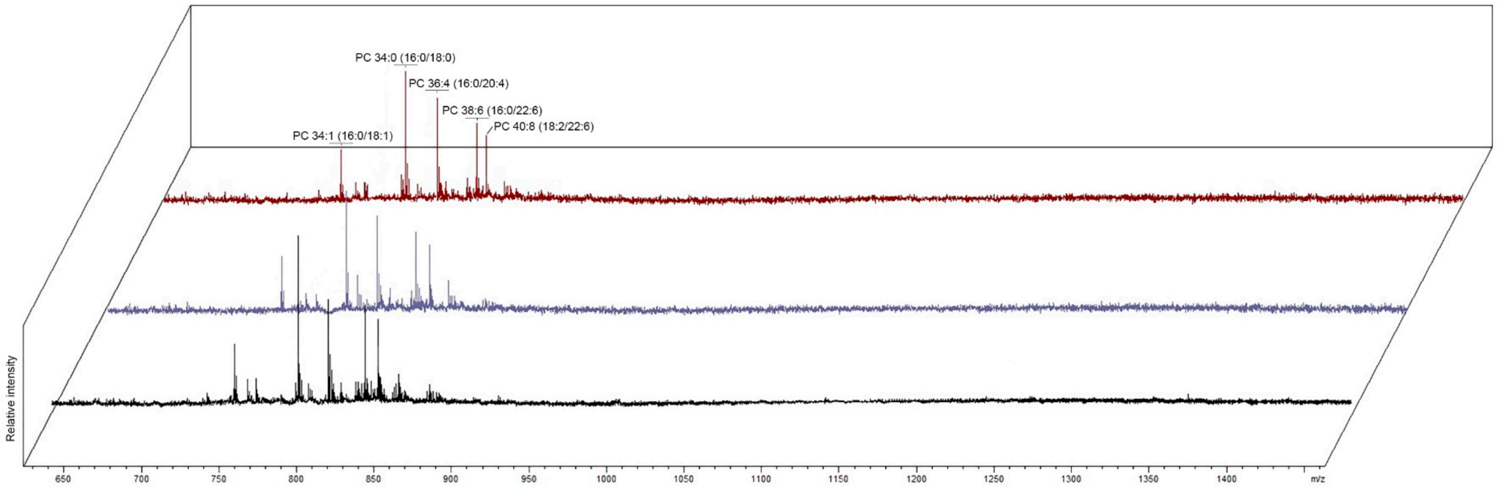

Figure S1. Representative MALDI-MSI spectra of phosphatidylcholine (PC) species at Day 7. Representative mass spectra from brain tissue sections at Day 7 show the relative abundance of major phosphatidylcholine (PC) species across experimental groups. Prominent peaks corresponding to PC 34:1 (m/z ~760.5), PC 34:0 (m/z ~800.5), PC 36:4 (m/z ~820.5), PC 38:6 (m/z ~844.5), and PC 40:8 (m/z ~852.5) are indicated. Differences in peak heights reflect relative signal abundance, enabling visual comparison of dominant lipid species between groups. Day 7 was selected as a representative time point because it exhibited clear and consistent differences in lipid profiles between groups while preserving key features of disease progression.

Table S1. Putative identification of phosphatidylcholine (PC) species based on observed m/z values in MALDI-MSI analysis.

Observed m/z	Putative PC (sum composition)	Putative acyl composition	Putative adduct	Reference
760.5	PC 34:1	16:0/18:1	[M+H]+	Siangcham et al., 2015
800.5	PC 34:0	16:0/18:0	[M+K]+	Siangcham et al., 2015
820.5	PC 36:4	16:0/20:4	[M+Na]+	Sugiura et al., 2009
844.5	PC 38:6	16:0/22:6	[M+Na]+	Sugiura et al., 2009
852.5	PC 40:8	18:2/22:6	[M+Na]+	Chansela et al., 2012
